# The effects of a high-intensity exercise bout on landing biomechanics post anterior cruciate ligament reconstruction: a quasi-experimental study

**DOI:** 10.1186/s13102-021-00263-7

**Published:** 2021-04-07

**Authors:** Ahmad Dhahawi Alanazi, Katy Mitchell, Toni Roddey, Aqeel M. Alenazi, Msaad M. Alzhrani, Ahmed M. Almansour, Alexis Ortiz-Rodriguez

**Affiliations:** 1 Department of Physical Therapy, College of Applied Medical Sciences, Majmaah University, Al-Majmaah, 11952 Saudi Arabia; 2 Texas Woman’s University, School of Physical Therapy, Houston, TX USA; 3 Department of Rehabilitation Sciences, Prince Sattam Bin Abdulaziz University, Alkharj, Saudi Arabia; 4 School of Physical Therapy. University of the Incarnate Word, San Antonio, TX USA

**Keywords:** Soccer, Fatigue, High-intensity exercise, Landing biomechanics, ACL reconstruction

## Abstract

**Background:**

We aimed to examine the effect of a high-intensity exercise bout on landing biomechanics in soccer players who underwent anterior cruciate ligament reconstruction (ACLR) and non-injured soccer players during a soccer-specific landing maneuver.

**Methods:**

Eighteen soccer players who underwent ACLR and 18 normal soccer players were enrolled in this investigation (ACLR group; age, 26.11 ± 3.95 years; body mass index, 23.52 ± 2.69 kg/m^2^; surgery time, 5 ± 3.30 years: control group; age, 25.83 ± 3.51 years; body mass index, 24.09 ± 3.73 kg/m^2^, respectively). Participants were evaluated during the landing maneuver before and after carrying out the high-intensity exercise bout using the Wingate test. The intensity of the exercise was defined as a blood lactate accumulation of at least 4 mmol/L. The dependent variables included sagittal-plane kinematics and kinetics of the ankle, knee and hip joints, and electromyography activity of the gastrocnemius, hamstrings, quadriceps, and gluteus maximus.

**Results:**

On 2 × 2 analysis of variance, none of the dependent variable showed significant exercise×group interactions. Regardless of group, significant main effects of exercise were found. Post-exercise landing was characterized by increased flexion of hip (*p* = 0.01), knee (*p* = 0.001), and ankle joints (*p* = 0.002); increased extension moments of hip (*p* = 0.009), knee (*p* = 0.012), and ankle joints (*p* = 0.003), as well as decreased quadriceps activity (*p* = 0.007).

**Conclusion:**

At 1 year or more post-ACLR, the effect of the high-intensity exercise bout on landing biomechanics is not expected to differ from that experienced by healthy soccer players.

## Background

Soccer is characterized by high-intensity activities in which a number of very different physical maneuvers are performed throughout the match such as running, jumping, heading, and changing directions [1, 2]. The overall distance covered during a soccer game is nearly 10 km, with 0.65 km being covered while sprinting [1]. Previous researchers have reported that the total distance covered is shorter in the second half than in the first half of the match [2]. A similar observation was reported regarding high-intensity actions, jumping ability, and sprinting after the soccer game, suggesting that fatigue developed during the game results in reduced physical performance [3, 4]. Consequently, substantial research interest has been invested in evaluating the influence of fatigue on lower extremity biomechanics.

Previous studies have reported that neuromuscular fatigue may induce several biomechanical changes that might increase the risk of anterior cruciate ligament (ACL) injuries during landing [5, 6]. Several impairments persist following ACL reconstruction (ACLR), including altered landing patterns, abnormal postural control, and neuromuscular deficits, which may magnify the detrimental effects of fatigue [7, 8]. However, previous results in this direction have been inconsistent, and the effect of fatigue in lower extremity injuries remains conflicting. In a study of 10 male ACLR recipients and 11 non-injured healthy males exposed to a general fatigue protocol to evaluate landing biomechanics during single-limb landing, the researchers found that fatigue induced many biomechanical changes in the ACLR limb, including decreased knee flexion and adduction moments [9]. Nevertheless, most biomechanical changes in the ACLR limb were also observed in the uninvolved limb, as well as in the limbs of healthy participants. On the other hand, some researchers found that fatigue-induced deficits were greater among ACLR individuals than in healthy individuals by the former exhibiting a significant reduction in hip extensor strength following the fatigue protocol [10].

Sagittal plane variables have been reported by previous investigators as predisposing factors contributing to the mechanism of ACL injury [11–15]. Landing with greater lower extremity joint flexion may facilitate greater energy absorption by muscles; therefore, decreasing the load to the passive component of the knee joint [16]. Previous researchers have found that limited sagittal plane motion was associated with greater knee valgus angles and decreased energy absorption by the hip and knee joints in female soccer players [17]. Those authors suggested that landing with limited sagittal plane motion may place individuals at higher risks of ACL injury. Moreover, other researchers showed that decreased hip flexion and increased external knee flexion moments were associated with greater risk of ACL injury [18].

Although some studies have investigated the effect of fatigue on kinematics, kinetics, and neuromuscular strategies following ACLR, such parameters have not been investigated in ACLR soccer players performing sports-specific landing maneuvers. Specifically, no study has compared kinematics, kinetics, and muscle activations between soccer players who underwent ACLR and healthy non-injured soccer players during landing before and after a high-intensity exercise bout. Landing maneuvers, such as landing after heading the soccer ball, are among the most common activities performed repeatedly throughout a soccer game. Landing after heading the soccer ball was selected in this study based on our previous investigation in which unplanned landing (landing after heading a soccer ball) exhibited increased injury predisposing factors compared with the forward jump [19]. It is unknown how the high-intensity exercise bout influences landing biomechanics during a soccer-specific landing maneuver in this population. The combination of the high-intensity exercise bout and the soccer-specific landing maneuver may further alter landing biomechanics, which may magnify the risk of ACL injury. Therefore, it is clinically important to evaluate the effects of a high-intensity exercise bout on landing biomechanics in soccer players with ACLR as this could contribute to design better rehabilitation programs that aim to prevent further injuries.

The purpose of the present investigation was to compare kinematics, kinetics, and muscle activations between soccer players who underwent ACLR and healthy non-injured soccer players during soccer-specific pre-exercise and post-exercise landings. We hypothesized that participants (in both groups) would demonstrate smaller flexion angles and greater extension moments (in the sagittal plane) and decreased muscle activations following the high-intensity exercise bout. Another hypothesis was made stating that decreased flexion angles, increased extension moments and decreased muscle activations are expected to be more prominent in the ACLR group.

## Methods

### Participants and study design

This quasi-experimental study was approved by our institution’s ethics review board (Dnr: 17902). Informed consent for participation was obtained from all subjects before enrollment. With an expected effect size (ES) of 0.30, α set at 0.05, and power at 0.80, a minimum sample size of 10 participants was deemed as necessary for each group [9]. The general inclusion criteria for this study were: current participation in soccer activities at recreational level and age between 18 and 35 years. The general exclusion criteria were: failure to execute the jump heading task, surgery of lower extremity or low-back; injury of the lower extremity during the 6 months leading up to the enrollment, self-reported pregnancy, ligament injuries of the lower extremity, other relevant conditions including neurological diseases, and bleeding disorders (e.g., hemophilia).

A sample of convenience including 18 soccer players (ACLR group; men: women, 8:10) who underwent ACLR (patellar tendon autograft, *n* = 10; hamstring autograft, *n* = 7; allograft, *n* = 1) was recruited for this investigation. Of 18 ACLR participants, 15 had unilateral ACLR (dominant leg, *n* = 7). The specific inclusion criterion for ACLR participants was to have undergone ACLR at least 1 year but not more than 10 years prior to study participation. The specific exclusion criterion for ACLR participants was having > 3 mm anterior tibial translation difference between the left and right knees, as measured using a knee arthrometer (MEDmetric Corp. San Diego, CA, USA). The control group consisted of 18 non-injured soccer players sex-matched to the ACLR participants (Table 1).

**Table 1:** Anthropometric data

Anthropometric measure	ACLR (*n* = 18)	Control (*n* = 18)	*P*-value
	Mean ± SD	Mean ± SD	
Age, years	26.11 ± 3.95	25.83 ± 3.51	0.82
Height, m	1.70 ± 0.09	1.66 ± 0.05	0.20
Mass, kg	68.15 ± 9.64	66.88 ± 10.37	0.70
BMI, kg/m^2^	23.52 ± 2.69	24.09 ± 3.73	0.60
Playing time, hours/week	4.11 ± 3.42	3.77 ± 3.07	0.76
Lactate levels, mmol/L	11.92 ± 4.56	11.30 ± 5.40	0.71
Time since surgery, years	5 ± 3.30	NA	NA

*ACLR* Anterior cruciate ligament reconstruction; *BMI* Body mass index; *NA* Not applicable; *SD* Standard deviation

### Instrumentation

Fifteen retro-reflective markers were attached over body landmarks based on the Vicon Plug-in Gait biomechanical model (version 2; Vicon Motion Systems Ltd., Denver, CO, USA). Body landmarks included both anterior superior iliac spines, at the second sacral vertebra, bilaterally at the lateral femoral epicondyles, bilaterally at mid-distance between the greater trochanter and lateral femoral epicondyle, at the lateral malleoli, bilaterally at mid-distance between the lateral femoral epicondyle and lateral malleolus, at the calcaneal tuberosities, and at the second metatarsophalangeal joints. The trials were recorded using a Vicon motion analysis system consisting of 10 digital cameras (240-Hz sampling rate) and four AMTI (Advanced Mechanical Technology Inc., Watertown, MA, USA) force platforms (1920-Hz sampling rate). The equipment was calibrated according to the manufacturer’s recommendations and a static trial was conducted before each data collection session.

A Trigno Wireless EMG system (Delsys Inc., Boston, MA, USA) was used to measure muscle activity of the gluteus maximus, vastus lateralis, rectus femoris, vastus medialis, biceps femoris and semitendinosus, and lateral gastrocnemius in both legs. These muscles were selected due to their major role in dissipating the impact forces during dynamic functional tasks. While a more flexed knee position during landing may be facilitated by increased concentric action of the hamstrings and gastrocnemius muscles, gluteus maximus and quadriceps muscles act eccentrically to control the flexion angles at the hip and knee joints, respectively [20–22]. Using double-sided tape, 14 pre-amplified wireless electrodes were placed bilaterally over the muscle belly, according to a well-established procedure [23]. Before placing the electrodes, the skin was cleansed using a cotton ball soaked in 70% isopropyl alcohol. To reduce movement artifacts, hypoallergenic tape was used to secure the electrodes in place during the functional tasks.

A portable Lactate Plus analyzer (Sports Resource Group Inc., Hawthorne, NY, USA) with a measuring range of 0.3–25 mmol/L of blood lactate was used for determining blood lactate concentration after the high-intensity exercise bout. Lactate accumulation levels of 4 mmol/L were considered as the anaerobic threshold [24]. The Lactate Plus analyzer is widely used for measuring blood lactate levels in clinical and laboratory settings, with excellent reliability (r = 0.99) in healthy males and concurrent validity (r = 0.97) against the reference Yellow Springs Instrument 2300 (YSI Inc., Yellow Springs, OH, USA) in men and women [25, 26].

A Just Jump System (Probotics Inc., Huntsville, AL, USA) was utilized to determine the maximum vertical jump height. The Just Jump System has been used to assess vertical jump height in many strength and conditioning studies, having very good reliability (ICC ≥ 0.87) and providing data highly correlated with measurements obtained using the reference three-camera motion analysis system (r = 0.96) [27, 28].

A KT-1000 Arthrometer (MEDmetric Corp., San Diego, CA, USA) was utilized to measure anterior tibial translation and any differences between both knees were noted. The KT-1000 has been frequently used to measure anterior tibial translation with mm-level precision in clinical setting involving ACL disruption and ACLR, with good reliability and validity [29, 30]. In particular, the reliability of KT-1000 was confirmed in healthy male subjects (ICC ≥ 0.84), providing a specificity of 0.72 and a sensitivity of 0.90 in patients with unilateral ACL deficiency [31, 32].

### Procedure

Anthropometric data were obtained from each participant. Then, each participant was instructed to perform a warm-up protocol consisting of 5 min of cycling at 40–60 rpm on a cycle ergometer, 10 half squats, and five continuous vertical countermovement jumps. To determine maximum vertical jump height and maximum long jump distance, each participant was instructed to perform three vertical jumps and three long jumps as far as possible. The highest vertical jump and the longest forward jump were documented for each participant. To familiarize the participants with the landing task evaluated in this study, each participant was given a demonstration of the functional task and instructed to perform two practice trials previously shown to provide good reliability of measurements (ICC ≥ 0.76) [33].

The landing task included a forward jump to head a soccer ball and land on the force platforms. The soccer ball was suspended from the ceiling at a distance equivalent to 40% of the participant’s maximum long jump from the starting point [19]. A detailed description about the landing task included in this study has been reported previously [19]. Participants were instructed to carry out four trials based on our initial reliability study that found four trials provided good reliability for all kinematics and kinetics parameters evaluated during the landing task (ICC ≥ 0.76).

The high-intensity exercise bout required the participants to perform a 30-s Wingate anaerobic protocol after reading a set of instructions meant to standardize the amount and type of verbal encouragement received throughout the Wingate protocol [34]. After a warm-up period of 2 min, the participant was asked to pedal as fast as possible for 30 s against a pre-determined resistance calculated as the subject’s weight multiplied by 0.090 kp [34]. Immediately after completing the Wingate protocol, blood samples were taken from the participant’s fingertip to determine the blood lactate concentration. The intensity level of the exercise bout was set at a lactate concentration of ≥4 mmol/L, which is recognized as the anaerobic threshold [24]. Participants who did not reach this level were instructed to perform an additional 30-s bout of pedaling. All participants reached the desirable level of exercise intensity within their first trial of the Wingate test (Table 1). Participants were then instructed to perform four trials of the landing maneuvers. In order to limit recovery from the intensity of the exercise throughout the post-exercise session, all trials were performed within 30 s of each other. Furthermore, participants were asked to continue performing squats while data were saved in the computer and the Vicon system was being prepared for the subsequent trials.

### Data reduction

All kinematic and kinetic data were synchronized and analyzed using Vicon Nexus 1.8 and the Polygon software, version 4.0 (Vicon Motion System Ltd.). The 3-dimensional trajectory of retro-reflective markers, from which joint angles were derived, was filtered through a fourth-order no-lag Butterworth filter at a frequency of 10 Hz. An initial reliability investigation demonstrated that majority of kinematics and kinetics parameters provide good test-retest reliability only on sagittal plane mechanics (ICC ≥ 0.83). Therefore, the kinematics and kinetics parameters evaluated during the landing maneuver focused on the sagittal plane mechanics. These parameters included peak ankle dorsiflexion angle, peak plantar flexion moment, peak knee flexion angle, peak knee extension moment, peak hip flexion angle, and peak hip extension moment. Kinetic data were filtered through a fourth-order no-lag Butterworth filter at a frequency of 50 Hz. The Cardan-Euler representation (XYZ sequence) and inverse dynamic methods were used to calculate the joint angles and moments, respectively. Joint moments were normalized to body mass (newton meter per kilogram), expressed in the coordinate system of the distal segment, and reported as external moments. Joint angle and moment data were exported to Excel (Microsoft Corp., Redmond, WA, USA) and then transferred to SPSS (IBM Inc., Chicago, IL USA) for analysis. For each parameter, the peak value was defined as the greatest value recorded between the moment the participant landed on the force plates and the moment the participant achieved maximum knee flexion. For each participant, the average of the peak values of both limbs was calculated for each parameter, and this average value was used for statistical analysis.

EMG data were time-synchronized to kinematic and kinetic data and analyzed using Polygon software. For each muscle, the mean and maximum signals recorded between the initial contact and the moment of maximum knee flexion were exported to Excel. The average EMG data were normalized by dividing the mean signals by the maximum signals for each muscle. This approach (dynamic normalization procedure) is frequently utilized for normalizing EMG data acquired during dynamic maneuvers and helps reduce between and within subject variability [19, 35–37]. The normalized data for the vastus medialis and lateralis, and rectus femoris, and biceps femoris and semitendinosus were averaged to constitute the quadriceps and hamstring muscle groups, respectively. Then, the average normalized data of both limbs were exported for statistical analysis.

### Statistical analysis

The kinematic, kinetic, and EMG data were screened for normality and outliers using the Kolmogorov-Smirnov test and box plot test, respectively. For each anthropometric parameter, an independent t-test was conducted to check for differences between groups at baseline. A 2 × 2 mixed analysis of variance (group×exercise) was performed for each dependent variable. Group (ACLR vs. control) served as the between-subjects factor, while exercise (pre-exercise vs. post-exercise) served as the within-subject factor. To reduce bias associated with type-I errors, the α-level was adjusted to 0.05/3 (i.e., 0.0167). Further adjustment of the α-level was made for simple effects and follow-up comparisons (to 0.0167/2 or 0.0083). Effect sizes (ES) and power were calculated. Effect sizes were calculated using F-ratios based on the following formula: $r=\sqrt{\frac{F\ \left(1,{df}_R\right)}{F\ \left(1,{df}_R\right)+{df}_R}}$ [38]. SPSS version 23 (IBM Inc., Chicago, IL USA) was utilized to conduct all analyses.

## Results

All kinematic, kinetic, and EMG data met the assumptions of normality and outliers. None of the anthropometric parameters differed significantly between the groups (Table 1). There were no significant exercise×group interactions for any outcome measure. However, there were significant main effects of exercise regardless of group. Specifically, post- exercise landing was characterized by greater hip flexion (F_1,34_ = 7.24, *p* = 0.01, ES = 0.41, β = 0.74), greater knee flexion (F_1,34_ = 12.16, *p* = 0.001, ES = 0.51, β = 0.92), and greater ankle dorsiflexion (F_1,34_ = 10.97, *p* = 0.002, ES = 0.49, β = 0.89) (Table 2). Additionally, post- exercise landing was characterized by significantly greater hip extension moments (F_1,34_ = 7.71, *p* = 0.009, ES = 0.42, β = 0.77), greater knee extension moments (F_1,34_ = 7.04, *p* = 0.012, ES = 0.41, β = 0.73), greater ankle plantarflexion moments (F_1,34_ = 10.38, *p* = 0.003, ES = 0.48, β = 0.87), and decreased quadriceps activity (F_1,34_ = 8.18, *p* = 0.007, ES = 0.44, β = 0.79) regardless of group assignment (Tables 2 and 3).

**Table 2:** Kinematics and kinetics data

Variable	Pre-exercise	Post-exercise	*P*-value		
	Mean ± SD	Mean ± SD	ME-G (β)	ME-E (β)	Interaction (β)
Kinematics, °					
Hip Flexion					
ACLR	69.96 ± 15.57	79.33 ± 18.53	0.15 (0.29)	0.011^*^	0.03 (0.58)
Control	65.38 ± 19.54	66.25 ± 21.45			
Knee Flexion					
ACLR	74.17 ± 12.86	83.22 ± 15.72	0.34 (0.15)	0.011^*^	0.04 (0.53)
Control	73.62 ± 11.9	75.83 ± 12.73			
Dorsiflexion					
ACLR	23.25 ± 6.07	26.11 ± 5.65	0.62 (0.07)	0.002^*^	0.11 (0.34)
Control	24.93 ± 4.22	25.91 ± 2.41			
Kinetics, Nm/kg					
Hip Extension moment					
ACLR	2.27 ± 0.65	3.15 ± 1.15	0.02 (0.64)	0.009^*^	0.11 (0.35)
Control	2.11 ± 0.86	2.34 ± 0.61			
Knee Extension moment					
ACLR	1.80 ± 0.43	2.37 ± 0.93	0.07 (0.44)	0.012^*^	0.07 (0.06)
Control	1.56 ± 0.78	1.99 ± 0.75			
Plantarflexion moment					
ACLR	0.79 ± 0.35	1.07 ± 0.35	0.08 (0.40)	0.003^*^	0.58 (0.08)
Control	0.66 ± 0.39	0.86 ± 0.40			

*ACLR* Anterior cruciate ligament reconstruction; *β* Power; *ME-G* Main effect for group; *ME-E* Main effect for exercise; *SD* Standard deviation. ^*^Statistically significant (*p* < 0.0167)

**Table 3:** Electromyography data

Variable	Pre-exercise	Post-exercise	*P*-value		
	Mean ± SD	Mean ± SD	ME-G (β)	ME-E (β)	Interaction (β)
Muscle Activity					
Gluteus maximus					
ACLR	73.60 ± 17.26	70.93 ± 18.36	0.99 (0.05)	0.83 (0.05)	0.52 (0.09)
Control	71.54 ± 15.11	72.90 ± 17.67			
Quadriceps					
ACLR	79.18 ± 8.91	74.66 ± 3.67	0.02 (0.61)	0.007^*^	0.94 (0.50)
Control	84.62 ± 6.28	80.32 ± 12.70			
Hamstrings					
ACLR	60.86 ± 6.29	65.19 ± 14.66	0.07 (0.42)	0.17 (0.26)	0.67 (0.06)
Control	67.07 ± 6.91	69.38 ± 14.14			
Gastrocnemius					
ACLR	56.26 ± 13.46	59.48 ± 10.77	0.08 (0.41)	0.44 (0.11)	0.04 (0.54)
Control	66.00 ± 5.59	59.16 ± 11.08			

*ACLR* Anterior cruciate ligament reconstruction; *β* Power; *ME-G* Main effect for group; *ME-E* Main effect for exercise; *SD* Standard deviation. ^*^Statistically significant (*p* < 0.0167)

## Discussion

The purpose of this study was to evaluate the effect of high-intensity exercise bout on kinematic, kinetic, and muscle activations during a soccer-specific landing maneuver performed by ACLR and healthy, non-injured soccer players before and after the Wingate anaerobic protocol. Our findings do not support our hypothesis that participants were expected to exhibit to decrease flexion angles during the post-exercise landing when compared to the pre-exercise landing. Specifically, after the high-intensity exercise bout, participants landed with significantly increased hip flexion, knee flexion, and ankle dorsiflexion (Table 2). These findings are consistent with previous observations that fatigue increases flexion angles at the hip and knee as well as ankle dorsiflexion during a single-leg drop landing performed by non-injured male and female athletes [39, 40]. On the contrary, others reported decreased hip flexion, knee flexion, and ankle dorsiflexion during single-leg drop landing [41]. Taken together, these findings indicate that fatigue induces alterations in landing mechanics by either increasing or decreasing the lower extremity joint angles. In other words, individuals may respond to the effect of fatigue by either increasing or decreasing flexion angles (a stiffer or softer landing technique) to absorb the landing impact. While stiff landing techniques are thought to increase the risk of lower extremity injuries, soft landings are suggested to facilitate the distribution of forces that act during landing [42].

In the present study, which focused on recreational soccer players, the participants adopted a softer landing technique following the high-intensity exercise bout, potentially indicating decreased power of the gluteus maximus and quadriceps muscles, which act eccentrically to decrease flexion angles at the hip, knee, and ankle joints [43]. Previous researchers have reported that increased eccentric contraction of gluteus maximus and quadriceps muscles may result in a more extended position during landing [20–22, 44]. Upon examining the EMG data recorded during the landing task, we found that only the normalized signal for quadriceps muscles was significantly lower post-exercise than pre-exercise in both groups (Table 3). Therefore, the decreased activation of the quadriceps muscles might, in part, explain the soft landing technique utilized by the participants in our study following the Wingate anaerobic exercise.

Our kinetics data indicated that post-exercise landing was characterized by greater internal hip extension, knee extension, and plantar flexion moments than noted for pre-exercise landing (Table 2), which might be explained by increased hip flexion, knee flexion, and ankle dorsiflexion angles [45]. At the hip and knee joints, greater flexion angles may lead to greater external flexion moments, which must be counteracted by greater internal extension moments [45]. At the ankle joint, greater dorsiflexion angles may lead to greater external dorsiflexion moments, which must be counteracted by greater internal plantar flexion moments [45]. Thus, the findings of the present investigation indicate that the mechanical demand on the hip extensor, knee extensor, and plantar flexor muscles increased following the Wingate anaerobic protocol.

Our present results do not support the second hypothesis, namely that alterations induced by the high-intensity exercise bout would be more pronounced in the ACLR group than in the control group. In this investigation, the ACLR group and control group demonstrated similar alterations in landing mechanics and neuromuscular performance in response to the Wingate anaerobic protocol. This finding is in agreement with previous observations that the ACLR and control groups demonstrated similar kinematics and kinetics parameters during a single-leg landing task after completing a fatigue protocol that consisted of 10 bilateral squats to 90° of knee flexion [9]. However, another study reported greater hip extensor strength loss in the ACLR group compared to the control group in response to a fatigue protocol that included a 20-min anaerobic exercise on the treadmill [10]. In this context, the results of our investigation indicate that, at 1 year or more post-ACLR, high-intensity exercise-induced changes in landing biomechanics are expected to be comparable to those experienced by healthy, non-injured individuals.

Previous researchers suggested that increased flexion angles of the hip and knee joints (soft landing) may reduce the loading on the ACL during landing [42]. In the present investigation, participants in both groups altered their landing mechanics by increasing hip flexion, knee flexion and ankle dorsiflexion angles during the post-exercise landing. This may indicate that athletes may change their landing technique in response to a fatigue protocol. The adopted landing mechanics observed in this study (soft landing) may help the participants efficiently attenuate the external impact forces during landing and therefore reduce the ACL loading.

Some limitations in the present investigation should be taken into consideration when interpreting these results. The results of this study can be generalized to healthy recreational soccer players and soccer players with ACLR (around 5 years post-surgery). Changes observed as main effects were largely driven by changes in the ACLR group. This may indicate that soccer players in the ACLR group do in fact display differences to healthy soccer players; however, these differences were not observed due the small sample size. Also, evaluating the sagittal plane only without the medio-lateral and transverse axes data might limit the usefulness of these results as most ligamentous knee injuries have a triplanar mechanism of injury. Differences between men and women were not examined due to the small sample size in the current study. Furthermore, the type of ACLR techniques (allograft and autograft) varied across the ACLR group, and not all ACLR participants had been treated by the same orthopedic surgeon. The inherent limitations of EMG measurements and the calculation of joint moments during dynamic functional tasks might have influenced the results of the study. Additionally, different filter cut-off frequencies used for kinetics and kinematics may produce artifacts in joint moments. The landing phase used in this study might pose a limitation in determining differences in landing styles specifically at initial contact. Moreover, the high-intensity exercise bout (Wingate anaerobic protocol) used in the current study might not sufficiently reflect the intensity of the dynamic maneuvers that soccer players usually experience during a real soccer match.

## Conclusion

The results of this investigation indicated that the high-intensity exercise bout induced alterations in landing biomechanics, but these alterations did not differ significantly according to the ACLR history. Following the high-intensity exercise bout, individuals with ACLR showed changes in landing biomechanics that were comparable to those noted in healthy, non-injured individuals.

## Data Availability

The datasets analyzed during the current study are available from the corresponding author on reasonable request.

## Abbreviations

ACL: Anterior cruciate ligament
ACLR: Anterior cruciate ligament reconstruction
EMG: Electromyography
S.D.: Standard deviations
ES: Effect sizes
β: Power
ME-G: Main effect for group
ME-E: Main effect for exercise
